# Reduced hippocampal volume in adolescents with psychotic experiences: A longitudinal population-based study

**DOI:** 10.1371/journal.pone.0233670

**Published:** 2020-06-03

**Authors:** Ana Calvo, Darren W. Roddy, Helen Coughlan, Ian Kelleher, Colm Healy, Michelle Harley, Mary Clarke, Alexander Leemans, Thomas Frodl, Erik O’Hanlon, Mary Cannon

**Affiliations:** 1 Dept. of Psychiatry, Royal College of Surgeons in Ireland, Dublin, Ireland; 2 Faculty of Health Sciences, Universidad Internacional de la Rioja (UNIR), Madrid, Spain; 3 Department of Child and Adolescent Psychiatry, Hospital General Universitario Gregorio Marañón School of Medicine, Universidad Complutense, IiSGM, CIBERSAM, Madrid, Spain; 4 Trinity College Institute of Neuroscience, Trinity College Dublin, Dublin, Ireland; 5 Image Sciences Institute University Medical Center Utrecht, Utrecht, the Netherlands; 6 Department and Hospital of Psychiatry and Psychotherapy, Otto von Guericke University Mageburg, Mageburg, Germany; 7 German Center for Neurodegenerative Diseases (DZNE), Site Magdeburg, Magdeburg, Germany; King's College London, UNITED KINGDOM

## Abstract

**Aims:**

Smaller hippocampal volumes are among the most consistently reported neuroimaging findings in schizophrenia. However, little is known about hippocampal volumes in people who report psychotic experiences. This study investigated differences in hippocampal volume between young people without formal diagnoses who report psychotic experiences (PEs) and those who do not report such experiences. This study also investigated if any differences persisted over two years.

**Methods:**

A nested case-control study of 25 adolescents (mean age 13.5 years) with reported PEs and 25 matched controls (mean age 13.36 years) without PEs were drawn from a sample of 100 local schoolchildren. High-resolution T1-weighted anatomical imaging and subsequent automated cortical segmentation (Freesurfer 6.0) was undertaken to determine total hippocampal volumes. Comprehensive semi-structured clinical interviews were also performed including information on PEs, mental diagnoses and early life stress (bullying). Participants were invited for a second scan at two years.

**Results:**

19 adolescents with PEs and 19 controls completed both scans. Hippocampal volumes were bilaterally lower in the PE group compared to the controls with moderate effects sizes both at baseline [left hippocampus p = 0.024 d = 0.736, right hippocampus p = 0.018, d = 0.738] and at 2 year follow up [left hippocampus p = 0.027 d = 0.702, right = 0.048 d = 0.659] throughout. These differences survived adjustment for co-morbid mental disorders and early life stress.

**Conclusions:**

Psychotic experiences are associated with total hippocampal volume loss in young people and this volume loss appears to be independent of possible confounders such as co-morbid disorders and early life stress.

## Introduction

One of the most consistent structural neuroimaging findings across all brain areas in schizophrenia research is that of smaller hippocampal volumes in patients with the disorder [1–3]. Meta-analyses of patients with schizophrenia also reveal that the hippocampus shows the largest volumetric difference when compared to other brain structures [4]. Interestingly, smaller hippocampal volumes are similarly reported in patients with first-episode psychosis and also patients in the At-Risk Mental State (a preclinical state indicating a likely conversion to a formal diagnosis of a psychotic disorder) [5]. These findings suggest a potential etiological role for this key temporal lobe structure in schizophrenia and other psychotic disorders.

The hippocampus is of particular interest in psychosis due to its location deep within the temporal lobe and its role as a key limbic hub. This complex structure processes important memory and spatial information and then transmits this encoded information throughout the brain, in particular to regions involved with emotional, behavioral and cognitive processing [6–8]. These regions, such as the cingulate and frontal cortices, hypothalamic areas and adjacent temporal structures are also known to be involved in psychosis. Changes in the expression and transmission of neurotransmitters such as GABA (γ-aminobutyric acid) and glutamate in the hippocampus have also been proposed as potential etiological disruptions in psychosis [9–12]. Such hippocampal abnormalities found in patients with psychosis are most likely multifactorial in origin, involving a complex combination of genetic predisposition [13], perinatal brain development [14] dose and duration of antipsychotic treatment, [15, 16] co-morbid mental disorders [17,18] and in particular exposure to early life stressors [19, 20].

Recent research has proposed the existence of an extended psychosis phenotype in the general population [21, 22]. Rather than psychotic disorders existing as discrete independent entities, it is suggested that psychosis may exist as a continuum of reality testing with varying levels of severity of psychotic experience blending along the continuum [23]. Between 8–17% of children and adolescents [21] and 7% of adults [22] in the general population report psychotic experiences (PEs) such as hallucinations and delusions but only a small proportion ever meet the stringent criteria for a diagnosable psychotic disorder. This extended psychosis phenotype model proposes that schizophrenia lies at the extreme end of a psychosis continuum with the majority of individuals experiencing less severe hallucinatory and delusional experiences with varying degrees of intact reality testing [24]. However, it is reported that individuals who describe PEs in early life are at increased risk of being diagnosed with a psychotic disorder such as schizophrenia [25, 26]. Interestingly, recent research has shown that individuals who report psychotic experiences are at risk of a wide range of mental disorders. Such disorders are just not limited to psychosis, however, and include other diagnosable psychiatric conditions such as depression and anxiety [27–29], as well as other poor mental health outcomes including suicidal behavior [30], poorer socio-occupational function [28, 31], and neurocognitive deficits [32, 33]. Although PEs appear common in the general population and are associated with serious sequelae, little is known about the brain changes, if any, in individuals with PEs. In particular, there has been no research regarding hippocampal volumes in young individuals who report PEs but do not meet the criteria for an identified psychotic disorder such as schizophrenia.

Psychotic disorders have consistently been associated with early life stressors such as neglect, sexual, physical and emotional abuse [34, 35]. Recent studies have also shown an association between childhood maltreatment or bullying and the extended psychosis phenotype [36, 37]. Although the majority of psychotic symptoms do not persist over time (longitudinal studies typically demonstrate persistence rates of between 15% to 25% [38, 39], research has shown that ongoing exposure to trauma increases the odds that psychotic symptoms will persist [40]. Of note, young people with persistent psychotic symptoms have been shown to have poorer clinical and functional outcomes [41].

To our knowledge, there has been no published longitudinal research to date examining hippocampal volumes and early life stress in young people with psychotic experiences. We hypothesize that adolescents with PEs will show reduced global hippocampal volume both at baseline and at follow-up compared to adolescents without PEs. We further hypothesize that a proportion of this hippocampal volume loss may be accounted for by a history of early life stress and/or by the presence of co-morbid mental disorders. To these ends, in this study, we investigated 1) if hippocampal volume differences exist between young people who report PEs and those who do not; 2) if these differences, if any, persist for 2 years through adolescence and 3) if these differences could be associated with co-morbid mental disorders and early adversity.

## Methods

### Participants

Between July 2007 and September 2010, 212 young people aged between 11–13 years were recruited from primary schools in Dublin and Kildare, Ireland [42]. All 212 participants were white and native English-speaking and attended for a semi-structured clinical interview and neurocognitive testing. For further details on the recruitment and interview assessments see Kelleher et al. [42]. A subsample of 100 of the original study participants also agreed to take part in a neuroimaging study. 25 of these adolescents reported psychotic experiences and constituted the Psychotic Experiences (PEs) group. From the remaining participants with brain imaging, 25 adolescents without psychotic symptoms were selected to match the PE group for age (at the time of scanning), gender and handedness to create the control group. All participants were naïve for both psychotropic medication and illicit substance use. Participants were followed up two years after the baseline neuroimaging scans and interviews.

### Clinical interviews

The Kiddie Schedule for Affective Disorders and Schizophrenia (K-SADS) is a well-validated, semi-structured research diagnostic interview for the assessment of current and lifetime DSM-IV mental disorders in children and adolescents [43]. Interviews with both the young person and a parent were carried out at baseline and repeated with the young person at 2-year follow-up. All interviews were carried out by trained interviewers with a background in mental health.

### Psychotic experiences

The psychosis section of the K-SADS contains screening questions designed to assess hallucinations and delusions [42]. If one of the screening questions elicited a positive response, further details were ascertained using the SOCRATES template. This allowed perceptual abnormalities and unusual thought content to be explored and documented in a systematic and comprehensive manner. The SOCRATES template is available for download at https://epubs.rcsi.ie/psychart/19/. The history was transcribed and discussed at a consensus meeting involving three investigators (MC, IK, and MH who are mental health professionals with expertise in psychosis). At this meeting a decision was also reached on whether the young person had reported a “definite” psychotic symptom.

#### Mental disorders

Diagnoses of any Axis 1 mental disorders at baseline interview were made using DSM-IV criteria. This was coded as a yes/no variable.

#### Early life stress (bullying)

Information on any history of bullying up to the time of the baseline interview was obtained during the clinical interview. Bullying by our definition constituted either/and physical or psychosocial intimidation, harm, or coercion. This was coded as a binary yes/no variable depending on whether the participant endorsed being a victim of bullying at any time in their life.

Ethical approval was obtained from the Medical Research Ethics Committee, Beaumont Hospital, Dublin, and the School of Psychology, Trinity College Dublin. Written parental consent and participant assent were obtained prior to the study.

### High-resolution anatomy imaging

180 axial high-resolution T1-weighted anatomical images (TE = 3.8 ms, TR = 8.4 ms, FOV 230 x 230 mm^2^, 0.898 x 0.898 mm^2^ in-plane resolution, slice thickness 0.9 mm, flip angle alpha = 8°) were acquired. The acquisition time was approximately 6.5 minutes. All scanning was conducted on the same scanner (Philips Intera Achieva 3.0 Tesla) at the Trinity College Institute of Neuroscience, Dublin.

### Imaging analysis

Cortical reconstruction and volumetric segmentation was performed with the Freesurfer 5.3 image analysis suite (http://surfer.nmr.mgh.harvard.edu/) [44, 45]. The technical details of these procedures are described in prior publications [46–51]. In particular, the hippocampal analysis tool from the developmental version of FreeSurfer (v6.0 2017) was utilized to calculate hippocampal subfield volumes. This features novel algorithms using Bayesian inference [52] and high-resolution ex-vivo MRI atlas data offering improved accuracy and reliability [53]. Total hippocampal volume estimates where calculated by combining all individual subfield measures to yield a global hippocampal measure. An estimated measure for total intracranial volume (eTIV) was obtained from the routine output of Freesurfer using the MRI-seg- stats tool [54]. This procedure applies a one-parameter scaling factor calculated during the normalization transformation to the standard MNI305 brain.

### Statistical analysis

Statistical analysis was performed with SPSS software (version 21) [55]. All hippocampal volume measures were initially investigated using the “Explore” function within SPSS to assess data normality and outlier identification. Systematic inspection of the data was then performed. Outliers were defined as volumes greater than 1.5 x interquartile (IQ) range and extremes as 3 x IQ range as per the standard SPSS boxplot summary output. The normality of the quantitative variables was studied through the Kolmogorov-Smirnov test [56], to determine the appropriate use of either parametric or non-parametric hypothesis tests. Since the data did meet the assumption of normal distribution, parametric tests were used in their analysis. All comparisons were performed for each hemisphere independently.

In our primary analysis: mixed-model repeated measures (MMRM) was used in order to explore the effects of Group x Time interaction between the Control and PE group for hippocampal volumes during the 2-year follow-up controlled for estimated Total Intracranial Volume (eTIV), Axis-1 disorders and Early Life Stress (Bullying). Within this analysis we also studied the inter- and intra-group differences during the 2-year follow-up.

Forward stepwise forced entry linear regression was performed to determine whether differences in hippocampal volume could be accounted for by co-morbid mental disorders or early adversity. The variable ‘Hippocampus’ (left and right at baseline and two years follow up) was used as a dependent variable and, as independent factors, we included the “group” variable (PE and Control) and eTIV in the first step and bullying and other diagnosis in the second step.

Effect sizes were determined to quantify the differences between groups. The effect size was calculated within each treatment condition and then subtracting the control group from the experimental group effect size. The effect size for each treatment condition is defined as the pre to post test change divided by the pre-test standard deviation. The magnitude of effect sizes can be described as small (d = 0.2), medium (d = 0.5) and large (d = 0.8) [57]. In addition, the effect size of the regression analyses were based on Cohen’s (1988) guidelines with *f*^2^≥ 0.02, *f*^2^≥ 0.15, and *f*^2^ ≥ 0.35 representing small, medium, and large effect sizes, respectively.

## Results

### Demographics

There were no significant differences between the PE group and the control group for age, gender or handedness (Table 1). At baseline the presence of mental disorders and early life stress were common in the PE group (Table 1). 60% of adolescents with PEs had been bullied compared with 24% in the control group. Similarly, 60% of adolescents with PEs had been diagnosed with an Axis 1 mental disorder at baseline compared with 28% in the control group.

**Table 1:** Sociodemographic and clinical data at baseline. All values are expressed as percentages (%) unless otherwise indicated.

	PE, n = 25	Controls, n = 25	p-value
Mean age (years), mean (SD)	13.5 (1.26)	13.36 (1.15)	0.642
Gender (Male)	8(32)	10(40)	0.765
Handedness (Right)	23(92)	24(96)	0.552
Co-morbid Axis 1 mental disorders	15(60)	7(28)	0.023
History of Bullying	15(60)	6(24)	0.009

SD, standard deviation; PE, adolescents reporting psychotic experiences.

76% percent of the sample returned for follow-up after two years. PE group (n = 19) and the Control group (n = 19).

### Hippocampal volumes

**Fig 1:**
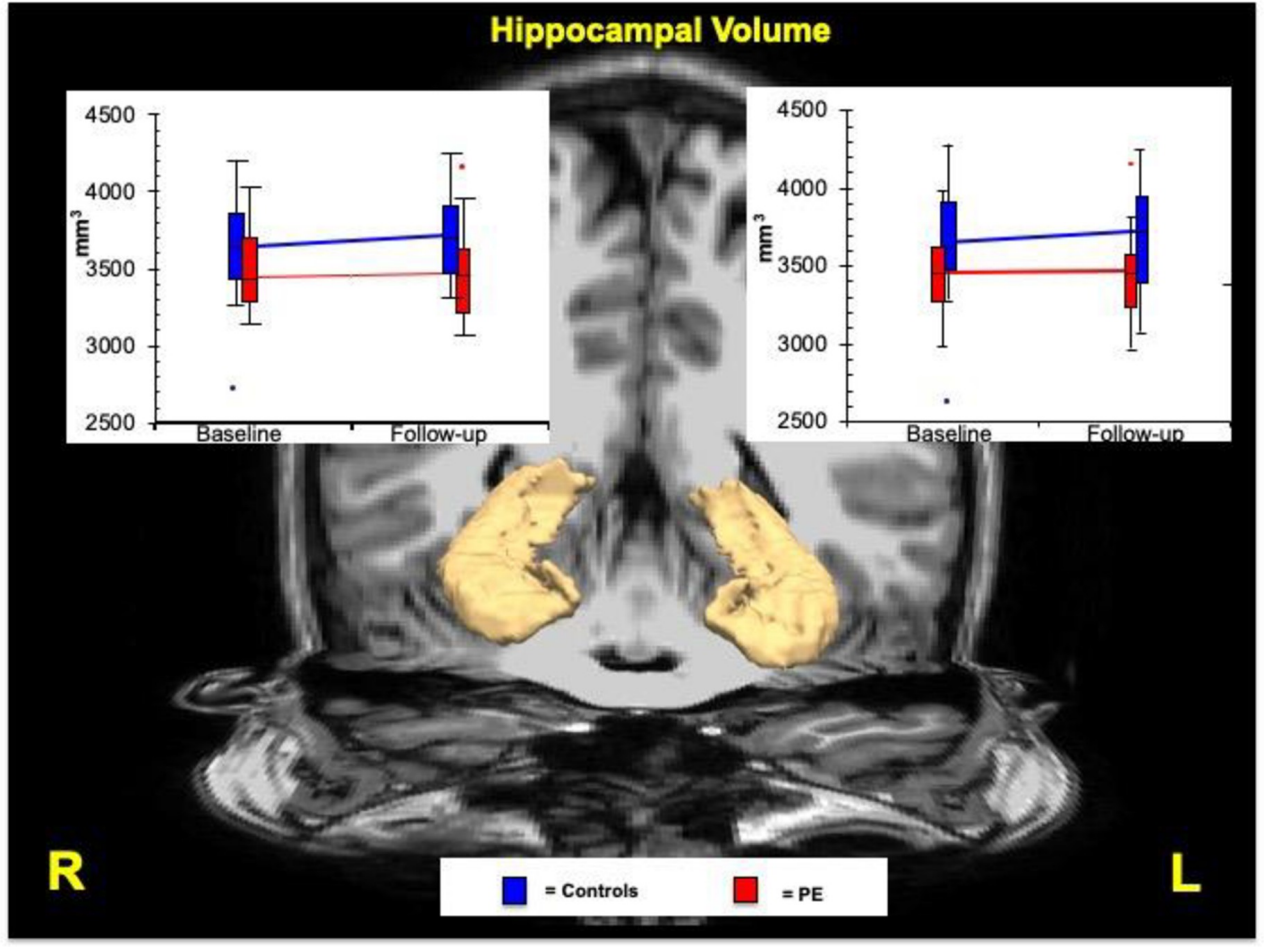
Patterns of change of hippocampal volume over 2 years follow-up between the PE and control group. Graphs show volumetric measures at baseline and follow-up in left and right whole hippocampus. Trendlines provide rates of volume change during the two time points between the PE and control group. Blue represents the control group and red represents the PE group. Outliers are shown as dots in both graphs. Volumes are shown in millimeters cubed. PE, adolescents reporting psychotic experiences.

A total of four outliers were removed; one from each group at both timepoints (see Fig 1). At baseline there was a main effect for Group on the left [F(1,76) = 10,182 (p = 0.002)] and right [F(1,76) = 9,488 (p = 0.003)] whole hippocampal volumes. No Group x Time interaction was found in either in the left (p = 0.944) or right (p = 0.735) whole hippocampal volumes.

In the inter-group analysis, significant differences were found between the PE and Control group at baseline in the left and right hippocampal volumes and these differences persisted at two-year follow-up (Table 2). The PE group had significantly smaller hippocampal volumes throughout the sample with a large effect size for both left (d = 1.266) and right (d = 0.768) hippocampus. Specifically, moderate effect sizes were seen between the groups at baseline for the left (d = 0,736) and right (d = 0,738) hippocampus, and similarly between the groups at two years for the left (d = -0,702) and right (d = -0,659) hippocampus.

**Table 2:** Mixed model analysis: Whole hippocampal volumes. Whole hippomcampal volumes were calculated throught the summation of all hippocampal outputs from using Freesurfer 6.0 and shown in millimeters cubed.

	PE hippocampal volumes (mm^3^)/(SEM)				Controls hippocampal volumes (mm^3^)/(SEM)				p-value (effect size)		
Structure	Baseline (n = 25)	95% CI	Follow-up (n = 19)	95% CI	Baseline (n = 25)	95% CI	Follow-up (n = 19)	95% CI	Baseline: time 1	Follow up: time 2	Overall effect size
Left	3462(55)	3346,964–3577.591	3466(59)	3311.197–3590.874	3652(56)	3534.530–3769.913	3727(62)	3532.229–3840.417	0.024 (0.736)	0.027 (0.702)	d = 1.266
Right	3448(57)	3330,873–3565.251	3475(61)	3307.085–3591.311	3651(58)	3532.264–3771.476	3723(64)	3505.952–3819.154	0.018 (0.738)	0.048 (0.659)	d = 0.768

CI, confidence interval; SEM, standard error of the mean.

In the intra-group analysis, no significant differences in hippocampal volumes were found within either the PE or group between baseline and two-year follow up.

In the fully adjusted model, significant differences were found in the left and right hippocampus between PE and Control group at baseline and these differences persisted at two-year follow up. The percentages of changes over time were different between the 2 groups (see Fig 1)

### Forward stepwise forced entry linear regression

Following linear regression, left and right hippocampal volumes at baseline and two years follow up were used as the dependent variables. The Group variable (PE and Control) and eTIV were included in the first step and Bullying and Axis-1 diagnosis as independent factors were included in the second step.

#### Left hippocampal volume linear regression

At baseline, Group and eTIV accounted for 30.1% of the variance in left hippocampal volume (F = 9.472, p = 0.0004, effect size f^2^ = 0.431 = large). Adding Bullying and Axis-1 diagnosis to the model did not significantly account for an additional proportion of the variance (Adjusted R^2^ changed: 2.5%, F = 0.774, p = .468, effect size f^2^ = 0.026 = small). In the second step, the beta co-efficients indicated that eTIV (Beta = 0.421, p = 0.002) and Group (Beta = -0.337, p = 0.023) contributed significantly to the model and were independently associated with left hippocampal volume but Bullying (Beta = 0.173, p = 0.227) and Axis-1 diagnosis (Beta = -0.067, p = 0.635) were not.

At two years follow up, Group and eTIV accounted for 50.9% of the variance in left hippocampal volume (F = 13.466, p = 0.0001, effect size *f*^2^ = 1.036 = large). Adding Bullying and Axis -1 diagnosis to the model significantly accounted for an additional proportion of the variance (Adjusted R^2^ changed: 17.2%, F = 6.464 p = 0.006, effect size *f*^2^ = 0.208 = medium). In the second step, the beta co-efficients indicated that eTIV (Beta = -0.618 p = 0.00002), Bullying (Beta = 0.458 p = 0.002) and Group (B = -0.38 p = 0.008), all contributed significantly and were independently associated as ordered with left hippocampal volume but Axis-1 diagnosis (Beta = -0.146 p = 0.260) was not.

#### Right hippocampal volume linear regression

At baseline, Group and eTIV accounted for 36.8% of the variance in right hippocampal volume (F = 12.790, p = 0.001, effect size *f*^2^ = 0.582 = large). Adding Bullying and Axis-1 diagnosis to the model did not significantly account for an additional proportion of the variance (Adjusted R^2^ changed: 4.5%, F = 3.27 p = 0.078, effect size *f*^2^ = 0.066 = small). In the second step, the co-efficients indicated that eTIV (Beta = 0.498, p = 0.001), Group (Beta = -0.336, p = 0.014), and Bullying (Beta = 0.261, p = 0.050) were independently associated with right hippocampal volume but Axis -1 diagnosis (Beta = -0.144, p = 0.265) was not.

In addition, at two years follow up, Group and eTIV accounted for 33.0% of the variance in right hippocampal volume (F = 6.416, p = 0.005, effect size *f*^2^ = 0.493 = large). Adding bullying and Axis-1diagnosis to the model did not significantly accounted for an additional proportion of the variance (Adjusted R^2^ changed: 8.0%, F = 1.622 p = 0.218, effect size *f*^2^ = 0.087 = small). In the second step, the co-efficients indicated that eTIV (B = 0.468, p = 0.008) was significantly independently associated with right hippocampal volume but Group (B = -0.348, p = 0.062), Bullying (B = 0.313, p = 0.085) and Axis -1 diagnosis (B = -0.084, p = 0.631) were not.

## Discussion

This study investigated hippocampal volumes in adolescents with psychotic experiences (PEs) and hippocampal volumes in adolescents without PEs at two time-points, two years apart. Hippocampal volumes were found to be smaller bilaterally in the young adults who reported PEs at both timepoints. These differences were not accounted for by differences in the rate of co-morbid mental disorders or early life stress between the groups.

While significant brain variations have been found to exist in individuals with diagnosable psychotic disorders, there has been limited research on the neurobiology of the extended psychosis phenotype. Our group has previously reported structural and functional differences in frontotemporal regions [58] and bilateral white matter differences in both frontotemporal and striatal regions in adolescents with PEs [58, 59]. Other recent population-based studies identified both global reduced gray matter volume and increased white matter volume, and, more specifically decreased hippocampal and amygdala volumes with structural and functional dysconnectivity in young adults with psychotic symptoms [60–63].

Adolescents with PEs have a four-fold increased risk of developing a psychotic disorder [25]. Smaller hippocampal volumes are one of the most replicated neuroimaging findings in patients with psychosis [64–66], implying an important role for this highly integrated limbic structure in the disorder. Our finding of bilateral smaller hippocampal volumes at baseline scan and also at two-year follow up in adolescents with PEs suggests that hippocampal volume may have a role as an early vulnerability marker for psychosis. The mean age of 13 years in our PE group at baseline scan suggests that smaller bilateral hippocampal volumes may already be present in late childhood in those at risk for psychosis.

A recent study reported that childhood trauma is associated with hippocampal and amygdala volume in first-episode psychosis and suggests that childhood traumatic experiences may contribute to the different brain morphology in individuals diagnosed with psychosis [67]. Previous studies found that environmental factors including early life stress may also be implicated in reduced hippocampal volume as has been shown in depression [68, 69]. Adverse early life environmental factors are thought to interact with an inherent genetic vulnerability to produce hippocampal change [69]. Similarly, another recent study suggests that the effect of childhood trauma on the risk of psychosis may be mediated through changes in hippocampal function [70].

In this study, we accounted for the possible moderating effect of a commonly reported early life stress, bullying. Studies vary, but between 11–21% of children relate being bullied in early life [71, 72]. Adolescents in our study reported marginally higher rates of bullying in controls (24%) and particularly higher rates in those who experienced psychotic symptoms (60%). The higher reported rates in controls may be due to local factors or potentially as a result of recent destigmatization campaigns run by the local health and social services in Ireland. These campaigns encourage young adolescents to report and talk about bullying and other experiences. The increased reported bullying in our cohort of adolescents who experienced psychotic symptoms is consistent with higher rates of bullying found in other studies of individuals in the extended psychosis phenotype. Although this study found that bullying may be associated with smaller hippocampal volumes, the association between PEs and hippocampal volume appears to be independent of the effects of bullying. Similarly, this study did not find an effect of Axis-1 disorders diagnosis on hippocampal volumes, over and above the experience of PEs. All participants were free of any psychotropic medication, including antipsychotics, negating a role for these medications in the smaller hippocampal volumes found in this study [14].

### Strengths and limitations

The strengths of this study include a well-described and well-matched sample of treatment and substance-naive adolescents. The use of a community-based sample rather than a hospital or clinic-based sample increases the generalizability of these results to other adolescent groups. 75% of the sample returned for repeat scanning on the same scanner two years later. Our analysis accounted for potential confounding by comorbid mental disorders and early life stress. In terms of limitations, we acknowledge that our sample is relatively small, localized to an Irish context and that our findings require replication in a larger and broader international sample. Also, this study only investigated bullying as a source of early life stress. It is entirely possible that other forms of abuse and neglect may reveal different relationships between hippocampal size and PEs in adolescents.

In conclusion, our findings of lower global bilateral hippocampal volumes in young people who report PEs suggest that smaller hippocampi at age 13 may be indicative of a broad psychosis phenotype. This study also found that young people who report PEs in early adolescence do not appear to recover hippocampal volumes to control levels after two years. These findings suggest a role for hippocampal volumes as potential biomarkers for psychosis later in life and hint at the benefits of early identification and treatment of young people who report PEs to alleviate hippocampal volume loss. This study also highlights the need for further neuroimaging and other biological research to elucidate the mechanism and relationship between smaller hippocampi in early adolescents with PEs and psychosis in later life.
